# Effect of enzyme‐assisted hydrolysis on protein pattern, technofunctional, and sensory properties of lupin protein isolates using enzyme combinations

**DOI:** 10.1002/fsn3.1286

**Published:** 2019-12-01

**Authors:** Katharina Schlegel, Katharina Sontheimer, Peter Eisner, Ute Schweiggert‐Weisz

**Affiliations:** ^1^ Emil Fischer Center Department of Chemistry and Pharmacy Friedrich‐Alexander‐Universität Erlangen‐Nürnberg Erlangen Germany; ^2^ Department Food Process Development Fraunhofer Institute for Process Engineering and Packaging IVV Freising Germany; ^3^ ZIEL ‐ Institute for Food & Health TUM School of Life Sciences Weihenstephan Technical University of Munich Freising Germany

**Keywords:** enzymatic hydrolysis, lupin protein isolate, protein functionality, sensory properties, sodium dodecylsulfate–polyacrylamide gel electrophoresis

## Abstract

The modification of lupin protein isolates (LPI) by means of enzymatic hydrolysis (*Lupinus angustifolius* cultivar Boregine) was performed with four enzyme preparations (Alcalase 2.4 L, Papain, Corolase 7089, and Neutrase 0.8 L) in a one‐ and two‐step process to determine the efficacy for the destruction of major IgE‐reactive polypeptides and the evaluation of the technofunctional and sensory properties of lupin protein hydrolysates. Combinations of Alcalase 2.4 L and Papain were most effective in the degradation of polypeptides in *L. angustifolius* as measured by sodium dodecylsulfate–polyacrylamide gel electrophoresis. The enzymatic hydrolysis of the LPI increased their technofunctional properties such as protein solubility, foam activity, and emulsifying capacity almost independently of the enzyme preparation used. The sensory results showed a significant increase in bitterness from 1.9 for LPI to 5.7 for the combination of Alcalase 2.4 L and Papain in one‐step process. The aroma attributes of the hydrolysates were very similar to untreated LPI. The results of this study show the possibility of enzymatic hydrolysis of LPI to destroy the major IgE‐reactive polypeptides that increase the technofunctional properties of the isolates and thus their use in human nutrition as food ingredients.

## Graphical abstract

Modification of lupin protein isolate (LPI) by enzymatic hydrolysis of LPIs (*Lupinus angustifolius* cultivar Boregine) was carried out with four enzyme preparations in a one‐step and two‐step process to determine the efficacy for the destruction of important IgE‐reactive polypeptides and the evaluation of the technofunctional and sensory characteristics of lupin hydrolysates. Combinations of Alcalase 2.4 L and Papain were most efficient in the destruction of polypeptides in *L. angustifolius* measured by sodium dodecylsulfate–polyacrylamide gel electrophoresis. Most of the proteolytic enzymes increased the technofunctional properties such as protein solubility, foam activity, and emulsifying capacity of untreated LPI. The aroma attributes of the hydrolysates were very similar to the LPI. The results of this study show the possibility of enzymatic hydrolysis of LPI to destroy the main IgE‐reactive polypeptides that increase the technofunctional properties of the isolates and therefore their use for human nutrition as food ingredient.

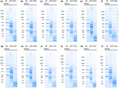

## 1 INTRODUCTION

The latest trends in human nutrition focus on the supply of protein‐enriched food products. The production of animal proteins mainly contributes to a disproportionate share of environmental impact, such as land use, air, and water quality, and greenhouse gas emissions (Eshel, Shepon, Makov, & Milo, 2014). A promising approach to reduce this impact on the environment could be achieved by the partial replacement of meat proteins by plant protein products in the human diet (Westhoek et al., 2014). Soy protein is one of the most important plant proteins, but has some disadvantages such as deforestation of rainforest or the use of genetically modified organisms. Thus, the search for alternative high‐quality plant protein sources is steadily increasing.

Lupins are widely grown in Europe. The high protein content, valuable technofunctional properties, and a well‐balanced sensory profile make lupins attractive for human nutrition (Arnoldi, Boschin, Zanoni, & Lammi, 2015; Bader, Oviedo, Pickardt, & Eisner, 2011). However, with the presence of lupin products in human nutrition, it has become clear that lupin proteins also contain an allergenic potential. The most abundant lupin seed proteins are storage proteins, which comprised the two major protein types α‐ (legumin‐like protein or 11*S* globulin) and β‐ (vicilin‐like protein or acid 7*S* globulin) conglutin, and γ‐ (basic 7S globulin), and δ‐ (2S sulfur‐rich albumin) conglutin in lower amounts (Duranti, Restani, Poniatowska, & Cerletti, 1981). Schlegel et al. (2019) described native α‐conglutin of *Lupinus angustifolius* cultivar Boregine are composed of low‐molecular‐weight (10–23 kDa), medium‐molecular‐weight (27–36 kDa), and high‐molecular‐weight (41–84 kDa) polypeptides and β‐Conglutin comprised of polypeptides with lower molecular weights (10, 13, 15, 16, and 18 kDa) and higher molecular weights polypeptides (27, 28, 31, 38, 46, 58, and 71 kDa) under reducing conditions. Goggin Mir Smith Stuckey and Smith (2008) observed a strong IgE reaction for polypeptides of β‐conglutin >40 kDa and a more weakly reaction for the proteins within the 25–31 kDa range in *L. angustifolius* L. Furthermore, β‐conglutin with a molecular weight of ~55–61 kDa has been designated Lup an 1 by the International Union of Immunological Societies (IUIS) allergen nomenclature subcommittee. Many reports propose a cross‐sensitization with other legumes such as soybean, pea, lentil, chickpea (Jappe & Vieths, 2010), and peanut (Faeste, Lovik, Wiker, & Egaas, 2004; Moneret‐Vautrin et al., 1999), probably due to structurally similar proteins including similar epitope regions (Jimenez‐Lopez et al., 2018).

Several attempts have been addressed to reduce the allergenic potential of food proteins to appeased allergic reactions in sensitive individuals (Chizoba Ekezie, Cheng, & Sun, 2018). One possible method is the inactivation of allergens by heat treatment, but this also has a considerable impact on food quality. Nonthermal technologies including pulsed light, high‐pressure processing, gamma irradiation, cold plasma technology, ultrasonication, and pulsed electric fields were also described (Chizoba Ekezie et al., 2018; Meinlschmidt, Ueberham, et al., 2016), but most of these methods do not achieve the complete inactivation of allergens or have not been adequately studied.

Another promising approach for inactivating allergens is enzymatic hydrolysis and fermentation of the proteins. Based on soy, extensive or mild protein hydrolysis can be used to prepare hypoallergenic foods (Lqari, Pedroche, Girón‐Calle, Vioque, & Millán, 2005). Moreover, several studies showed a great potential of protein hydrolysis for modifying functional properties, such as protein solubility, foaming, and emulsifying capacity (Chabanon, Chevalot, Framboisier, Chenu, & Marc, 2007; Hall, Jones, O'Haire, & Liceaga, 2017; Lqari et al., 2005; Meinlschmidt, Schweiggert‐Weisz, Brode, & Eisner, 2016; Meinlschmidt, Sussmann, Schweiggert‐Weisz, & Eisner, 2016; Purschke, Meinlschmidt, Horn, Rieder, & Jäger, 2018). However, protein hydrolysis can also affect the sensory properties of the products by producing a bitter taste which inhibits their use as a food ingredient (Spellman, O'Cuinn, & FitzGerald, 2004). Most of the studies described in literature and mentioned above focus on soy proteins. There is no literature data available that describe attempts to reduce the allergenic potential of lupin protein. In a previous study, Schlegel et al. (2019) investigated the impact of single protease treatments on technofunctional and sensory properties as well as on the molecular weight distribution to estimate the reduction of the immunoreactivity in lupin protein isolate (LPI) and hydrolysates. The objective of the current study was to determine the effectiveness of different protease combinations for the degradation of major IgE‐reactive polypeptides in *L. angustifolius* cultivar Boregine and the evaluation of the technofunctional characteristics of lupin hydrolysates. The influence of hydrolysis on the sensory attributes of LPIs was also investigated.

## 2 MATERIALS AND METHODS

### 2.1 Raw materials and chemicals

Lupin (*L. angustifolius* L. cultivar Boregine) seeds were purchased from Saatzucht Steinach GmbH & Co KG. The sources and properties of the used enzymes are listed in Table 1.

**Table 1:** Sources and properties of the enzymes used in this study

Enzyme	Type	Biological source	Supplier
Alcalase 2.4 L FG	Serine endopeptidase	*Bacillus licheniformis*	Novozymes A/S)
Papain	Cysteine endopeptidase	Papaya (*Carica* sp.) latex	AppliChem GmbH
Corolase 7089	Metallo‐ and serine endopeptidase	*Bacillus subtilis*	AB Enzymes GmbH
Neutrase 0.8 L	Metallo endopeptidase	*Bacillus amyloliquefaciens*	Novozymes A/S

### 2.2 Preparation of LPI

Lupin protein isolate was prepared from *L. angustifolius* L. cultivar Boregine. Seeds were dehulled and the hulls were separated by air‐sifting. Dehulled kernels were passed through a roller mill and the resulting flakes were de‐oiled in *n*‐hexane. Flakes were suspended in 0.5 M HCl at a 1:8 ratio. After extraction for 1 hr, the suspension was separated with a decanter centrifuge (5,600 *g*, 4°C, 1 hr) and supernatant was discarded. The acid pre‐extracted flakes were dispersed in 0.5 M NaOH (pH 8.0) at a 1:8 w/w ratio and stirred for 1 hr at room temperature. The suspension was separated (5,600 *g*, 4°C, 1 hr) and aliquots of 0.5 M HCl were added to the supernatant at room temperature to facilitate the protein precipitation at a pH of 4.5. The precipitated proteins were separated by centrifugation at 5,600 *g* for 130 min and then neutralized (0.5 M NaOH), pasteurized (70°C, 10 min) and spray dried.

### 2.3 Enzymatic hydrolysis of LPI

For the enzymatic hydrolysis of LPI, four enzyme preparations were used (Table 1) based on previous studies where promising results were achieved in the degradation of α‐conglutin and β‐conglutin in LPI (Schlegel et al., 2019). Reaction conditions (50°C and pH 8.0) were chosen according to Meinlschmidt, Schweiggert‐Weisz, et al. (2016). Hydrolysis experiments were carried out with the enzyme combinations shown in Table 2 in a 4 L thermostatically controlled reaction vessel. Therefore, the protein isolate was dispersed with an Ultraturrax (IKA‐Werke GmbH & Co. KG.) for 1 min at 5,000 rpm in deionized water at a protein concentration of 5% (w/w) and adjusted to 50°C and pH 8.0 with 3 M NaOH prior to enzyme addition. Hydrolysis was performed either as one‐step or two‐step process leading to 12 combinations of two or three enzymes (Table 2) according to Meinlschmidt, Schweiggert‐Weisz, et al. (2016) with some modifications.

**Table 2:** Combinations of protease preparations for LPI hydrolysis in one‐step and two‐step process

	Hydrolysis ID	Number of enzymes	Endoprotease (E/S %)			
			Alcalase 2.4 L	Papain	Corolase 7089	Neutrase 0.8 L
One‐step	OS 1	2	0.5		0.5	
	OS 2	3	0.5		0.5	0.5
	OS 3	3	0.5		0.5	1.0
	OS 4	2	0.5			0.5
	OS 5	2	0.5	0.2		
	OS 6	2		0.2	0.5	
	OS 7	3	0.5	0.2	0.5	
	OS 8	3	0.5	0.2		0.5
Two‐step	TS 1^a^	2	0.5 (1)		0.5 (2)	
	TS 2^a^	2	0.5 (1)			0.5 (2)
	TS 3^a^	2	0.5 (1)	0.2 (2)		
	TS 4^a^	2		0.2 (1)	0.5 (2)	

Abbreviation: E/S, Enzyme to solution ratio.

a LPI was hydrolyzed in two‐step process in the first stage for 1 hr with one enzyme (1) and after 1 hr the second enzyme (2) was added and hydrolysis was continued.

The one‐step process was carried out with eight different enzyme combinations. The enzyme preparations were added simultaneously to the vessel and hydrolyzed for 4 hr. Aliquots were taken after 2 and 4 hr. For the two‐step process, four different enzyme combinations were selected. The first enzyme preparation was incubated for 1 hr. Subsequently, the second enzyme preparation was added for another 4 hr. Aliquots were taken after 2 and 5 hr. During hydrolysis, the suspension was continuously stirred at controlled pH and temperature. To avoid further hydrolysis, the reaction was stopped by heating the protein suspension to 90°C for 20 min, cooled down to room temperature and neutralized with 3 M HCl (pH 7.0). Control LPI dispersions (no enzyme addition) were prepared under the same conditions and inactivation treatment. Samples were frozen at −50°C and lyophilized (BETA 1‐8; Martin Christ Gefriertrocknungsanlagen GmbH). Each hydrolysis experiment was performed twice.

### 2.4 Chemical composition

The protein content was determined according to the Dumas combustion method AOAC 968.06 using a protein calculation factor of N × 5.8 according to Mosse, Huet, and Baudet (1987). The dry matter was analyzed according to AOAC methods 925.10 in a TGA 601 thermogravimetric system (Leco Corporation) at 105°C.

### 2.5 Protein analysis

#### 2.5.1 Degree of hydrolysis

The degree of hydrolysis (DH) was quantified using the o‐phthaldialdehyde (OPA) method with serine as the standard as previously described by Nielsen, Petersen, and Dambmann (2001).

#### 2.5.2 Molecular weight distribution

The molecular weight distribution of LPI and its hydrolysates were determined by sodium dodecylsulfate–polyacrylamide gel electrophoresis (SDS‐PAGE) modified according to Laemmli (1970). SDS‐PAGE was performed in a vertical electrophoresis cell (Bio‐Rad Laboratories). LPI and LPI hydrolysates were applied at a protein equivalent of 10 µl sample per lane on a precast 4%–20% stain‐free polyacrylamide gel (Bio‐Rad Laboratories). Precision Plus Protein Unstained Standard with molecular weight of 10–250 kDa (Bio‐Rad Laboratories) run alongside as size markers, and the protein subunits were visualized using a Gel Doc™ EZ Imager system (Bio‐Rad Laboratories). The molecular weight distribution was determined using Image Lab software (Bio‐Rad Laboratories).

### 2.6 Technofunctional properties

#### 2.6.1 Protein solubility

Protein solubility (%) of LPI and its hydrolysates was determined in duplicate over the pH range of 4.0–9.0 following the method of Morr et al. (1985).

#### 2.6.2 Foaming properties

Foaming activity was determined in duplicate as recommended by Phillips, Haque, and Kinsella (1987). A 5% (w/w) protein solution (100 ml) at pH 7 and room temperature was whipped for 8 min in a Hobart 50‐N device (Hobart GmbH). The increase in volume after whipping was used to calculate the foam activity. The foam density (g/L) was measured by weighing a selected amount of foam volume and was defined as a ratio of foam volume to foam weight. The percentage leftover of foam volume after 1 hr was described as foaming stability (%).

#### 2.6.3 Emulsifying capacity

Emulsifying capacity of 1% (w/w) sample solution was determined at pH 7.0 in duplicate according to the method described by Wang and Johnson (2001) using a Titrino 702 SM titration system (Metrohm GmbH & Co. KG) at a constant rate of 10 ml/min until a phase inversion. The volume of oil needed to achieve the phase inversion was used to calculate the emulsifying capacity (ml oil per g sample).

### 2.7 Sensory analysis of protein hydrolysates

Sensory analysis was determined as previously described by Schlegel et al. (2019). Briefly, all samples were presented to the panel in plastic cups. Panelists (*n* = 10) were first required to record the retronasal aroma and taste attributes. The retronasal aroma attributes were rated on a scale from 0 (no perception) to 10 (strong perception) by each panelist. The taste attributes and trimeric astringent perception were also rated on a scale from 0 (no perception) to 10 (strong perception) with a nasal clamp by each panelist.

### 2.8 Statistical analysis

Results are expressed as mean ± standard deviation. Data were analyzed using one‐way analysis of variances (ANOVA) and means were generated and adjusted with Tukey's honestly significant difference post hoc test to determine the significance of differences between samples, with a threshold of *p* < .05. Statistical analysis was performed with Matlab R2018a for Windows (MathWorks).

## 3 RESULTS AND DISCUSSION

Alcalase 2.4 L and Papain showed the most effective results in depletion of major IgE‐reactive polypeptides in *L. angustifolius*. Furthermore, technofunctional properties of Alcalase 2.4 L, Papain, Corolase 7089, and Neutrase 0.8 L hydrolysates were improved and sensory attributes of the hydrolysates were very similar to the LPI.

The protein content of LPI and its proteolytic hydrolysates was about 92% and the dry matter was about 90%.

### 3.1 Degree of hydrolysis

The degree of hydrolysis (DH) was monitored to get—together with SDS‐PAGE analysis—a first indication of the size reduction of the proteins in order to estimate the reduction of the allergenic potential of the lupin proteins. The results are shown in Table 3. Untreated LPI had a DH value of 0.9%. Among the hydrolysates obtained after the one‐step process, the DH of OS 4 (Alcalase 2.4 L + Neutrase 0.8 L) increased to the highest of 14.0% after 4 hr, followed by OS 3 (Alcalase 2.4 L + Corolase 7089 + Neutrase 0.8 L), OS 2 (Alcalase 2.4 L + Corolase 7089 + Neutrase 0.8 L), OS 5 (Alcalase 2.4 L + Papain), and OS 1 (Alcalase 2.4 L + Corolase 7089) with DH values of 13.6%, 13.6%, 13.3%, and 12.7%, respectively. All of these hydrolysates were combination treatments with Alcalase 2.4 L, confirming the very effective lupin protein degradation by this preparation. In this previous study, a high DH value of 9.05% after 2 hr for the single Alcalase 2.4 L treatment in comparison to other enzyme preparations applied (DH in the range of 2.38%–6.90%) was shown. In comparison, the efficacy of Alcalase 2.4 L could be further improved within the enzyme combinations as all DH values after 2 hr of hydrolysis showed higher values than 9.1% with a maximum of 12.2% (Sample OS 5). Alcalase 2.4 L combinations with Papain (OS 7) and Papain and Neutrase 0.8 L (OS 8) with DH values of 10.4% for both after 4 hr hydrolysis were less efficient than other Alcalase 2.4 L combinations. OS 6 (Papain + Corolase 7089), the combination of Papain and Corolase, showed the lowest DH value of 3.1% after 4 hr hydroylsis.

**Table 3:** Degree of hydrolysis (DH) (%) of hydrolyzed LPI by different protease treatments

Hydrolysis ID	Degree of hydrolysis (%)			
	Time			
	0 hr	2 hr	4 hr	5 hr
OS 1	0.9 ± 0.1^a^	11.0 ± 0.4^c,d^	12.7 ± 0.4^c^	
OS 2	0.9 ± 0.1^a^	11.5 ± 0.1^d^	13.6 ± 0.2^d^	
OS 3	0.9 ± 0.1^a^	11.5 ± 1.2^d^	13.6 ± 0.4^d^	
OS 4	0.9 ± 0.1^a^	11.7 ± 0.3^d^	14.0 ± 0.3^d^	
OS 5	0.9 ± 0.1^a^	12.2 ± 1.5^d^	13.3 ± 0.3^c,d^	
OS 6	0.9 ± 0.1^a^	3.8 ± 0.8^a^	3.1 ± 0.34^a^	
OS 7	0.9 ± 0.1^a^	8.7 ± 0.2^b^	10.4 ± 0.5^b^	
OS 8	0.9 ± 0.1^a^	8.5 ± 0.1^b^	10.4 ± 0.2^b^	
TS 1	0.9 ± 0.1^a^	9.5 ± 0.5^b,c^		12.3 ± 0.2^c^
TS 2	0.9 ± 0.1^a^	9.2 ± 1.1^b^		11.9 ± 0.1^b^
TS 3	0.9 ± 0.1^a^	9.4 ± 0.1^b,c^		12.0 ± 0.1^b,c^
TS 4	0.9 ± 0.1^a^	3.1 ± 0.2^a^		5.3 ± 0.1^a^

Note

The data are expressed as mean ± standard deviation (*n* = 4). Values followed by different letter in a column indicate significant differences between groups (*p* < .05).

Among the hydrolysates obtained by the two‐step process, TS 1 (Alcalase 2.4 L + Corolase 7089) resulted in the highest DH value of 12.3% after 5 hr, followed by TS 2 (Alcalase 2.4 L + Neutrase 0.8 L) and TS 3 (Alcalase 2.4 L + Papain) with DH values of 11.9% and 12.0%, respectively. Similar to the one‐step process, all of these hydrolysates were prepared with enzyme combinations containing Alcalase 2.4 L. Similar to the one‐step process (OS 6, Papain + Corolase 7089), the enzyme combination of Papain and Corolase 7089 (TS 4) resulted in the hydrolysate with the lowest DH value with 5.3% after 5 hr. Schlegel et al. (2019) as well as Meinlschmidt, Sussmann, et al. (2016) described low DH values of hydrolysates obtained after Papain hydrolysis. The reason for low DH values could be due to the interaction between the released cysteine residues during hydrolysis with the cysteine endopeptidase Papain and the OPA reaction components, which reacted to an unstable, weakly fluorescence product, and distorted detection (Chen, Scott, & Trepman, 1979). The treatments of the LPI with the one‐step process were able to achieve higher DH values after 4 hr than the two‐step process after 5 hr. Similar results were observed by Meinlschmidt, Schweiggert‐Weisz, et al. (2016).

#### 3.1.1 SDS‐PAGE

**Figure 1:**
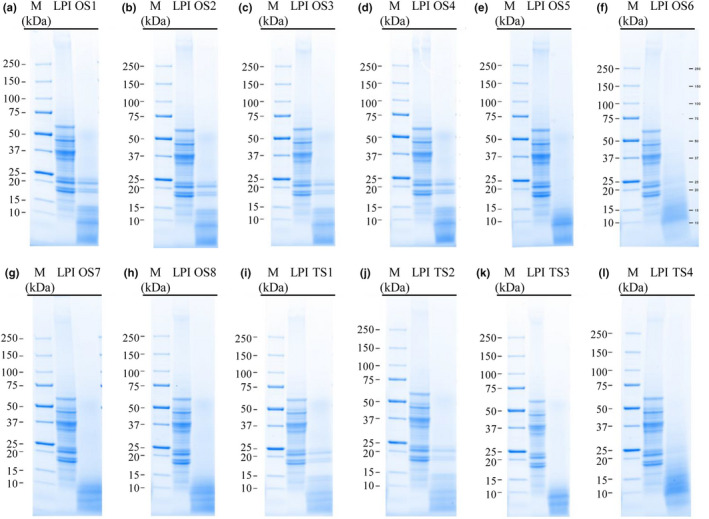
Peptide band profiles in LPI hydrolysates produced by treatment with different proteases combinations as determined by SDS‐PAGE under reducing conditions

Besides the determination of the DH, the molecular weight distribution of LPI and its hydrolysates was analyzed to get an indication of the integrity of the proteins. SDS‐PAGE results indicated that all treatments hydrolyze the polypeptides into smaller fragments with molecular sizes below 23 kDa and thus degrade the polypeptides responsible for most IgE reactions (Figure 1). According to Goggin et al. (2008), polypeptides of β‐conglutin with molecular weights of 12–16 kDa, as present in all hydrolysates, showed no IgE reaction.

OS 5 (Alcalase 2.4 L + Papain), OS 7 (Alcalase 2.4 L + Papain + Corolase 7089), OS 8 (Alcalase 2.4 L + Papain + Neutrase 0.8 L) and TS 3 (Alcalase 2.4 L + Papain) (Figure 1e,g,h,k) proved to be the most effective enzyme combinations by decomposing the polypeptides into fragments below 13 kDa. All of the mentioned hydrolysates were enzyme combinations of Alcalase 2.4 L and Papain. Alcalase 2.4 L is a serine endopeptidase from *Bacillus licheniformis* in which serine acts as a nucleophilic amino acid at the active site of the enzyme and cleaves the peptide bonds in proteins. Papain is classified as cysteine endopeptidase with specific substrate preferences for bulky hydrophobic or aromatic residues. The SDS‐PAGE results suggest that the combinations of serine and cysteine endopeptidase are able to hydrolyze the α‐conglutin and β‐conglutin fractions of LPIs higher 13 kDa and thus, the combination of Alcalase 2.4 L and Papain is more effective than Alcalase 2.4 L and Papain in separate use (Schlegel et al., 2019). In this study, the authors determined the most extensive hydrolysis of LPI treatments with Alcalase 2.4 L and Papain by breaking the polypeptides to a molecular size below 23 kDa. SDS‐PAGE results of OS 6 (Alcalase 2.4 L + Papain + Corolase 7089) and TS 4 (Papain + Corolase 7089) cannot be correlated with the observations of DH. The DH following OS 6 and TS 4 treatments were relatively low with 3.12% of OS 6 and 5.33% of TS 4, respectively. Both hydrolysates were combinations containing Papain and Corolase 7089. The differences could be potentially—as described above—due to interactions between the cysteine residues released during hydrolysis with Papain (cysteine endopeptidase) and the OPA reaction components, which react to an weakly fluorescent product (Chen et al., 1979).

### 3.2 Effects of enzymatic hydrolysis on the technofunctional properties

#### 3.2.1 Protein solubility

**Figure 2:**
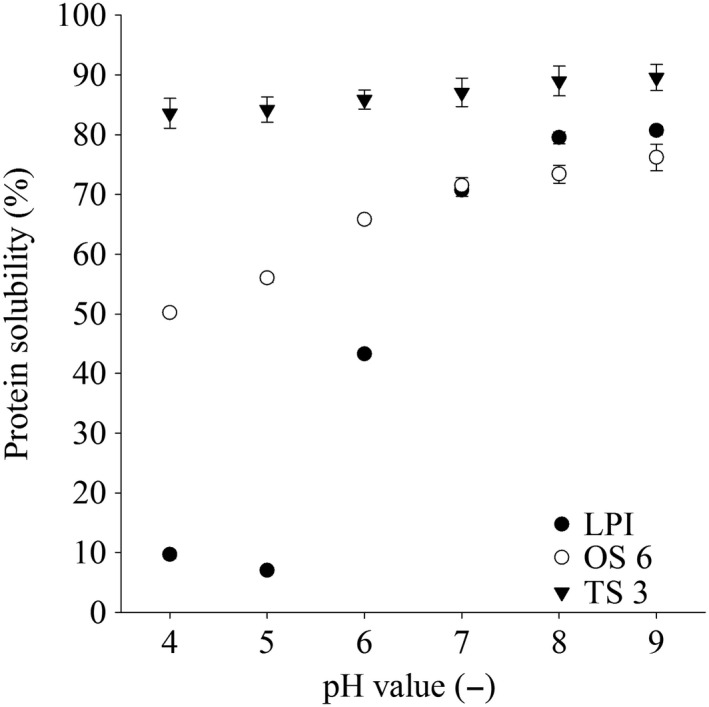
Solubility of LPI and LPI hydrolysates at pH range of pH 4.0 and pH 9.0. The data are expressed as mean ± standard deviation (*n* = 4)

The solubility of each lyophilized hydrolysate and LPI was determined as a function of pH in the range of 4.0 and 9.0 as shown in Figure 2 and Table 4. The maximum solubility of 80.7% of untreated LPI was detected at pH 9.0 and the minimum solubility of 7.0% at pH 5.0 (Figure 2), near the isoelectric point of lupin protein (Bader, Bez, & Eisner, 2011; Lqari et al., 2005; Piornos et al., 2015; Rodríguez‐Ambriz, Martínez‐Ayala, Millán, & Dávila‐Ortíz, 2005). Compared to untreated LPI, all hydrolysates exhibited a significantly higher solubility under acidic conditions. The increased solubility of the hydrolysates in acidic solutions compared to untreated LPI can be attributed to the fact that proteolysis generates short‐chain soluble peptides (Tsumura et al., 2005). The major influences on the solubility characteristics of proteins are hydrophobic interactions, which stimulate protein–protein interactions and lead to reduced solubility, and ionic interactions, which increase solubility by stimulating protein–water interactions (Kristinsson & Rasco, 2000). The enzymatic hydrolysis causes a significant structural change of the protein and cleaves it into smaller peptide units. The smaller molecule size and the new exposed ionizable groups increase the hydrophilicity and therefore the solubility of the hydrolysates (Gauthier, Paquin, Pouliot, & Turgeon, 1993; Qi, Hettiarachchy, & Kalapathy, 1997).

**Table 4:** Solubility of LPI and LPI hydrolysates at pH range of pH 4.0 and pH 9.0

Hydrolysis ID	Protein solubility (%)					
	pH 4	pH 5	pH 6	pH 7	pH 8	pH 9
LPI	9.7 ± 0.7^a^	7.0 ± 0.0^a^	43.3 ± 0.1^a^	70.7 ± 1.0^a^	79.5 ± 1.0^a,b,c^	80.7 ± 0.7^a,b,c^
OS 1	76.0 ± 0.8^d,e^	77.4 ± 1.5^c^	79.5 ± 1.4^c^	80.9 ± 1.6^c,d^	82.8 ± 0.4^b,c,d^	83.8 ± 0.6^b,c,d^
OS 2	61.5 ± 0.5^c^	82.2 ± 0.3^c^	83.1 ± 1.4^c,d^	84.8 ± 1.5^c,d,e^	83.6 ± 0.1^b,c,d^	84.8 ± 4.0^b,c,d^
OS 3	80.1 ± 3.1^e,f^	82.3 ± 5.3^c^	84.1 ± 5.4^d^	86.9 ± 4.5^d,e^	86.9 ± 5.6^c,d^	88.4 ± 5.2^c,d^
OS 4	81.4 ± 2.0^e,f^	84.5 ± 2.0^c^	87.2 ± 2.1^d^	87.6 ± 3.3^e^	89.2 ± 3.4^d^	90.1 ± 3.4^d^
OS 5	78.1 ± 1.4^d,e,f^	79.3 ± 1.0^c^	80.3 ± 1.1^c^	82.8 ± 1.4^c,de^	83.6 ± 1.4^b,c,d^	84.9 ± 2.4^b,c,d^
OS 6	50.2 ± 0.4^b^	56.0 ± 0.7^b^	65.8 ± 0.7^b^	71.5 ± 1.3^a^	73.4 ± 1.5^a^	76.2 ± 2.2^a^
OS 7	61.5 ± 0.5^c^	82.2 ± 0.3^c^	83.1 ± 1.4^c,d^	84.8 ± 1.5^c,d,e^	83.6 ± 0.1^b,c,d^	84.8 ± 4.0^b,c,d^
OS 8	79.1 ± 2.2^d,e,f^	76.6 ± 6.3^c^	82.4 ± 3.2^c,d^	84.3 ± 2.0^c,d,e^	88.1 ± 1.2^c,d^	89.7 ± 0.9^d^
TS 1	73.2 ± 6.9^d^	81.5 ± 6.6^c^	78.5 ± 4.8^c^	79.9 ± 4.9^b,c^	78.2 ± 6.6^a,b^	75.4 ± 1.7^a^
TS 2	78.6 ± 1.4^d,e,f^	82.8 ± 1.7^c^	86.4 ± 1.0^d^	87.8 ± 0.9^e^	86.3 ± 1.0^c,d^	87.9 ± 1.1^c,d^
TS 3	83.6 ± 2.5^f^	84.2 ± 2.1^c^	85.9 ± 1.6^d^	87.1 ± 2.4^d,e^	89.0 ± 2.5^d^	89.6 ± 2.2^d^
TS 4	54.3 ± 0.8^b,c^	59.9 ± 0.6^b^	69.7 ± 2.1^b^	73.4 ± 1.7^a,b^	77.1 ± 1.4^a,b^	79.7 ± 3.0^a,b^

Note

The data are expressed as mean ± standard deviation (*n* = 4). Values followed by different letters in a column indicate significant differences between groups (*p* < .05).

The TS 3 (Alcalase 2.4 L + Papain) hydrolysate showed the highest solubility of 83.6% at pH 4.0 compared to the other ones (Table 4). In contrast, the enzyme combination of Papain and Corolase 7089 has the lowest solubility of 50.2% in the one‐step process (OS 6) and 54.3% in the two‐step process (TS 4) at pH 4.0. Moreover, with the increase of pH value (pH > 5.0) solubility of LPI and its hydrolysates increased gradually. Schlegel et al. (2019) showed also a low solubility of hydrolysate LPI with Papain and Corolase 7089 as single enzymes. By increasing the concentration of Neutrase 0.8 L from 0.5% in OS 2 to 1.0% in OS 3, there is an increase in solubility at pH 4.0 from 61.5% to 80.1%. With the increase of pH value there was no significant difference between the combinations OS 2 (Alcalase 2.4 L + Corolase 7089 + Neutrase 0.8 L) and OS 3 (Alcalase 2.4 L + Corolase 7089 + Neutrase 0.8 L). The OS 4 (Alcalase 2.4 L + Neutrase 0.8 L) hydrolysate showed maximum solubility (90.1%) at pH 9.0.

**Figure 3:**
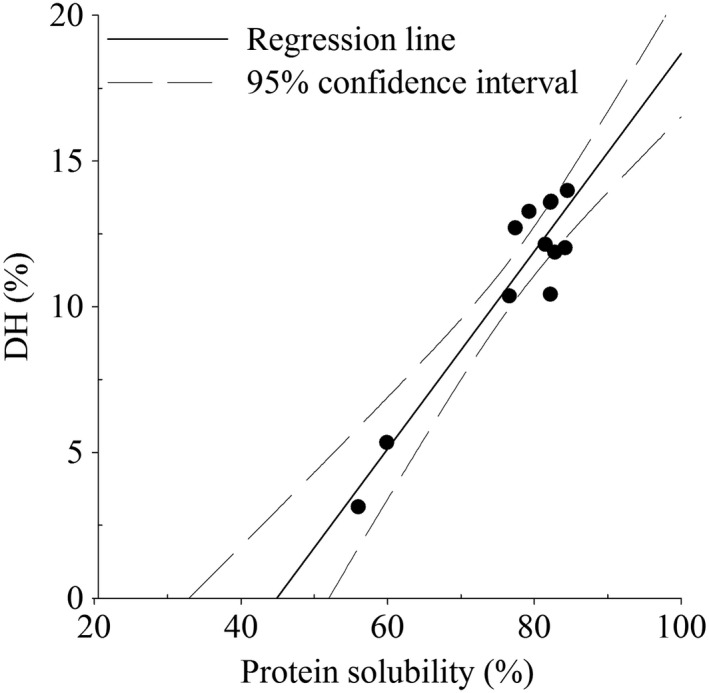
Degree of hydrolysis (DH) and protein solubility at pH 5.0 of LPI hydrolysates

Several studies described a correlation rate between solubility and the DH. This may be due to the fact that a higher DH showed a decrease in high‐molecular‐weight fractions, which exposed new ionizable groups and increased solubility. This study observed that the protein solubility of the hydrolysates with higher DH increased with a coefficient of correlation (*R* ^2^) of .88 (Figure 3).

#### 3.2.2 Foaming properties

The foaming properties (foam activity, stability, and density) of the hydrolysates are described in Table 5. All hydrolysates showed a significant increase in foaming activity compared to untreated LPI. TS 4 (Alcalase 2.4 L + Neutrase 0.8 L) and OS 6 hydrolysates (Papain + Corolase 7089) showed the highest foam activity with 2,721% and 2,660%, respectively, whereas OS 5 (Alcalase 2.4 L + Papain) showed the lowest with 2,198%. The authors Qi et al. (1997) observed that the increase of foaming activity in LPI hydrolysates reflects a change in protein structure that exposed the hydrophilic and polar groups to interactions with the aqueous environment. The foam stability among the various LPI hydrolysates showed significant variations within the OS 5 (Alcalase 2.4 L + Papain), OS 7 (Alcalase 2.4 L + Papain + Corolase 7089), OS 8 (Alcalase 2.4 L + Papain + Neutrase 0.8 L) and TS 3 (Alcalase 2.4 L + Papain) hydrolysates showing foam stability values of just 15%, 7%, 18% and 9%, respectively, whereas all other hydrolysates retained more than 79% stability after 1 hr. All hydrolysates with low stability values were combinations of Alcalase 2.4 L with Papain. El‐Adawy, Rahma, El‐Bedawey, and Gafar (2001) described that large peptides with flexible structures can stabilize foams. Stabilization of the air–water interface requires protein surface coverage, otherwise foam collapse occurs. The hydrolysis leads to the reduction of the protein surface coverage resulting in the collapse of the protein foams. This explanation is supported by our SDS‐PAGE profiles, which showed an extensive decrease in the molecular weight of the Alcalase 2.4 L and Papain. Stable foams could be formed from hydroysates with enzyme combinations of Alcalase 2.4 L with Corolase 7089 or Neutrase 0.8 L (OS 1, OS 2, OS 3, OS 4, TS 1, TS 2) or Papain with Corolase 7089 or Neutrase 0.8 L (OS 6, TS 4). The hydrolysis of the Alcalase 2.4 L and Papain (OS 5, OS 7, OS 8, TS 3) combinations may have caused smaller peptides than enzyme combinations containing only Alcalase 2.4 L or only Papain, which drastically reduced the stability of the hydrolysates. Interestingly, most of the hydrolysates showed excellent foam stability. LPI hydrolysates showed a significant decrease in foaming density compared to untreated LPI (Table 5). As expected, the hydrolysates OS 6 and TS 4 (Papain + Corolase 7089) showed a very low density of 29 and 33 g/L. This may be due to extensive hydrolysis and the resulting decrease in molecular weight, as shown by the SDS‐PAGE profiles.

**Table 5:** Technofunctional properties (foaming properties and emulsifying capacity) of LPI and LPI hydrolysates

Hydrolysis ID	Foam activity (%)	Foam stability at 1 hr (%)	Foam density (g/L)	Emulsifying capacity (ml/g)
LPI	980 ± 20^a^	84 ± 0^b,c^	98 ± 2^e^	620 ± 0^c^
OS 1	2,434 ± 155^b^	84 ± 5^b,c^	36 ± 2^b,c^	375 ± 14^b^
OS 2	2,268 ± 340^b^	91 ± 3^b,c^	39 ± 2^b,c,d^	370 ± 7^a,b^
OS 3	2,258 ± 190^b^	89 ± 3^b,c^	39 ± 4^b,c,d^	363 ± 4^a,b^
OS 4	2,226 ± 286 ^b^	79 ± 6^b^	38 ± 2^b,c,d^	370 ± 7^a,b^
OS 5	2,198 ± 262^b^	15 ± 4^a^	40 ± 1^c,d^	375 ± 14^b^
OS 6	2,660 ± 65^b^	81 ± 1^b,c^	29 ± 1^a^	625 ± 0^c^
OS 7	2,230 ± 319^b^	7 ± 6^a^	41 ± 4^c,d^	363 ± 18^a,b^
OS 8	2,370 ± 259^b^	18 ± 7^a^	39 ± 1^b,c,d^	358 ± 11^a,b^
TS 1	2,230 ± 2,282^b^	92 ± 3^c^	39 ± 2^b,c,d^	335 ± 0^a^
TS 2	2,308 ± 111^b^	91 ± 2^b,c^	43 ± 2^d^	350 ± 7^a,b^
TS 3	2,394 ± 312^b^	9 ± 1^a^	40 ± 1^c,d^	335 ± 0^a^
TS 4	2,721 ± 107^b^	86 ± 3^b,c^	33 ± 5^a,b^	608 ± 18^c^

Note

The data are expressed as mean ± standard deviation (*n* = 4). Values followed by different letters in a column indicate significant differences between groups (*p* < .05).

#### 3.2.3 Emulsifying capacity

As shown in Table 5, the emulsifying capacity of LPI (620 ml/g) was higher than most of the hydrolysates, with the exception of OS 6 and TS 4 (Papain + Corolase 7089) with 625 and 608 ml/g, respectively. Both were combinations of Papain with Corolase 7089. A direct correlation between the emulsifying capacity of proteins and their solubility was described in literature (El‐Adawy et al., 2001; Qi et al., 1997). Qi et al. (1997) described that more dissolved protein in an emulsion system will result in more protein in the interface between the oil phase and the continuous phase during emulsification. This correlation could not be observed in this study. Highly soluble hydrolysates, such as OS 4 (Alcalase 2.4 L + Neutrase 0.8 L) (87.6%) at pH 7.0 showed a decreased emulsifying capacity (370 ml/g) in comparison to the LPI (solubility 70.7% and emulsifying capacity 620 ml). However, the protein solubility of OS 6 (71.5%) and TS 4 (73.4%) (Papain + Corolase 7089) is similar to LPI (70.7%) at pH 7.0 and had a similar high emulsify capacity to LPI (620 ml/g) of 625 and 608 ml/g, respectively.

### 3.3 Sensory analysis of the protein hydrolysates

**Figure 4:**
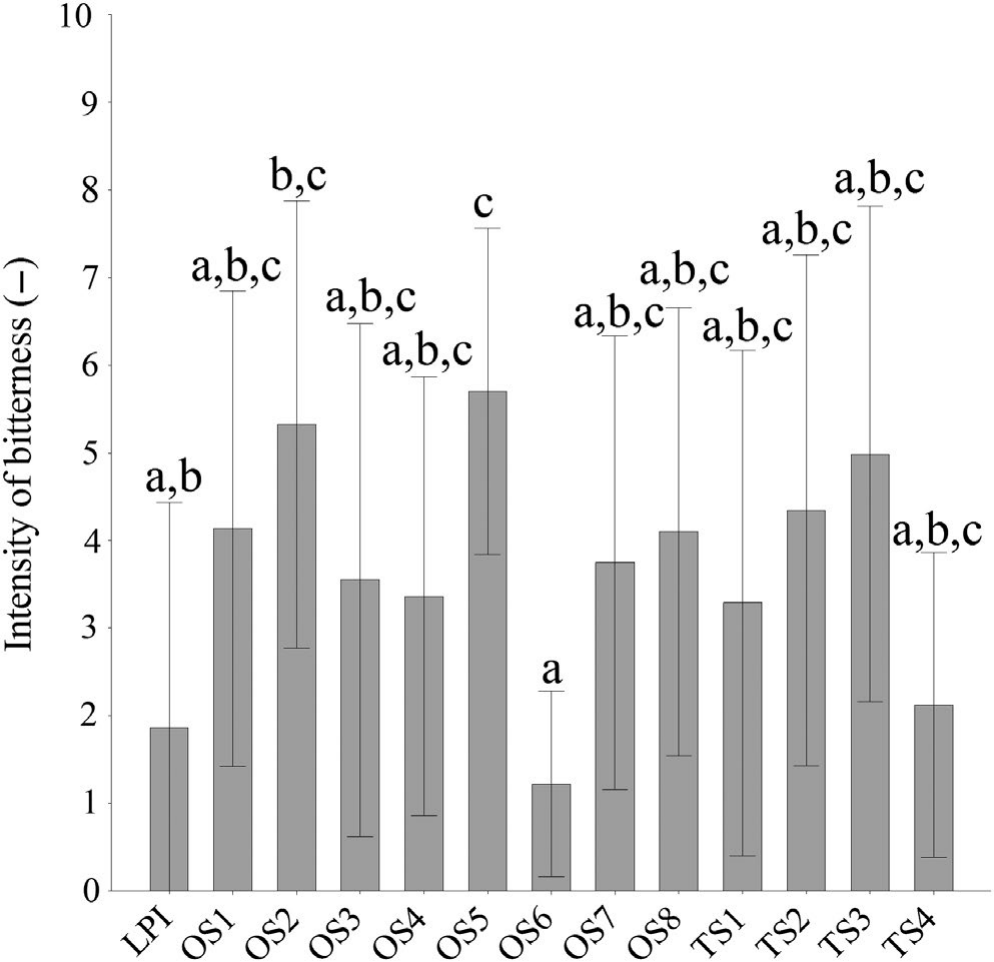
Intensities of bitterness of LPI and LPI hydrolysates. The data are expressed as the mean ± standard deviation scored on an unstructured 10 cm line between not noticeable at the left and very strong at the right, based on an evaluation by 10 panelists

The bitter taste of LPI and its hydrolysates was evaluated on a 10 cm continuous scale and the results are shown in Figure 4. Untreated LPI was judged with a bitterness intensity of 1.9. The bitter taste of the hydrolysates, with the exception of OS 5 (Alcalase 2.4 L + Papain) with a bitterness score of 5.7, was not significantly higher than that of untreated LPI. One of the most significant factors for bitterness is the hydrophobicity of peptides (Maehashi & Huang, 2009). Besides the hydrophobicity, the molecular size of the proteins might also play an important role for bitter perception. Matoba and Hata (1972) described that small hydrophobic peptides lead to the bitterness of protein hydrolysates. In this study, the SDS‐PAGE analysis showed that the extensive hydrolysis with the enzyme combination of Alcalase 2.4 L with Papain (OS 5) generated peptide chains of <15 kDa molecular weight and also created the most intense bitterness. Moreover, enzyme combinations containing no Alcalase 2.4 L preparations (OS 6 and TS 4, Papain + Corolase 7089) led to lower bitterness intensities of 1.2 and 2.1, respectively. Thus, enzyme combinations containing Alcalase 2.4 L resulted in the highest bitterness levels. Similar results were observed by Meinlschmidt, Schweiggert‐Weisz, et al. (2016) and Meinlschmidt, Sussmann, et al. (2016) for soy protein isolate. This is due to the bitter peptide generation of Alcalase 2.4 L, which are generally consisted of hydrophobic amino acid residues (Seo, Lee, & Baek, 2008). Schlegel et al. (2019) analyzed a bitterness score of 7.2 for a LPI hydrolysate treated by Alcalase 2.4 L. By enzyme combinations containing Alcalase 2.4 L, a lower maximum bitterness of 5.7 could be achieved. We could find a moderate correlation between DH values and bitter intensities of LPI hydrolysates with *R* ^2^ of .62. Hydrolysates with a high DH value led to higher bitterness intensity than hydrolysates with low DH values. Thus, the OS 6 and TS 4 hydrolysates (Papain + Corolase 7089) exhibited the lowest DH values of 3.14% and 5.33% and also the lowest bitter intensity of 1.2 and 2.1, respectively. All other hydrolysates had shown higher DH values and also high bitter intensity. Bitterness might correlate with DH and there is a positive correlation between bitterness and DH when DH values are low (Fu, Liu, Hansen, Bredie, & Lametsch, 2018; Newman et al., 2014).

The aroma attributes and salty taste of the individual LPI hydrolysates were further investigated in comparison to the untreated LPI. Untreated LPI showed a low perception of grassy, pea‐like, and cooked potato‐like aroma with 2.1, 1.3, and 1.7 and a medium strong perception of oatmeal‐like and fatty, cardboard‐like aroma with 4.7 and 4.0. TS 1 (Alcalase 2.4 L + Corolase 7089) showed a significantly lower perception of oatmeal‐like aroma (1.4) than untreated LPI (4.7), as shown in Table 6. The perception of fatty, cardboard‐like decreased significantly by OS 2 (Alcalase 2.4 L + Corolase 7089 + Neutrase 0.8 L), OS 4 (Alcalase 2.4 L + Neutrase 0.8 L), TS 2 (Alcalase 2.4 L + Neutrase 0.8 L) and TS 3 (Alcalase 2.4 L + Papain) with 1.1, 1.0, 1.1 and 1.5, respectively. The perception of cooked potato‐like showed a significantly low decrease by all hydrolysates (1.1) compared to untreated LPI (1.7). No significantly changes by all hydrolysates in the salty and astringent taste and the perception of grassy and pea‐like compared to untreated LPI were observed.

**Table 6:** Sensory profile (descriptive analysis) of nonhydrolyzed LPI and LPI hydrolysates

Hydrolysis ID	Salty	Astringent	Oatmeal‐like	Fatty, cardboard‐like	Grassy	Pea‐like	Cooked potato‐like
LPI	0.7^a^	0.8^a^	4.7^b^	4.0^b,c^	2.1^a^	1.3^a^	1.7^b^
OS 1	1.4^a^	3.6^a^	2.1^a,b^	1.4^a,b^	2.9^a^	2.6^a^	1.1^a^
OS 2	1.2^a^	3.7^a^	2.1^a,b^	1.1^a^	2.4^a^	2.1^a^	1.1^a^
OS 3	0.4^a^	2.6^a^	2.5^a,b^	1.6^a,b,c^	2.6^a^	2.9^a^	1.1^a^
OS 4	1.1^a^	2.4^a^	1.6^a^	1.0^a^	1.4^a^	1.6^a^	1.1^a^
OS 5	0.9^a^	1.7^a^	2.4^a,b^	1.2^a,b,c^	3.1^a^	2.9^a^	1.1^a^
OS 6	0.1^a^	0.6^a^	3.2^a,b^	1.6^a,b,c^	2.3^a^	1.2^a^	1.1^a^
OS 7	1.7^a^	2.3^a^	2.4^a,b^	1.5^a,b,c^	1.4^a^	1.2^a^	1.1^a^
OS 8	1.1^a^	3.1^a^	2.2^a,b^	1.2^a,b,c^	1.6^a^	1.6^a^	1.1^a^
TS 1	0.4^a^	1.2^a^	1.4^a^	1.5^a,b,c^	0.7^a^	2.4^a^	1.1^a^
TS 2	1.0^a^	1.5^a^	1.7^a,b^	1.1^a^	1.8^a^	2.0^a^	1.1^a^
TS 3	1.7^a^	1.3^a^	2.6^a,b^	1.5^a,b^	0.9^a^	1.8^a^	1.1^a^
TS 4	0.7^a^	0.6^a^	2.3^a,b^	1.0^a,b,c^	0.3^a^	0.7^a^	1.1^a^

Note

The data are expressed as the median values scored on an unstructured 10 cm line between not noticeable at the left and very strong at the right, based on an evaluation by 10 panelists (*n* = 10). Values followed by different letters in a column indicate significant differences between groups (*p* < .05).

## 4 CONCLUSION

The objective of this study was to evaluate the effectiveness of enzymatic hydrolysis with various protease combinations on the integrity of allergenic protein structures, technofunctionality, and sensory properties of LPI. The results showed the possibility of enzymatic hydrolysis with enzyme combinations to destroy the major IgE‐reactive polypeptides of *L. angustifolius*, while improving the technofunctional properties. According to the SDS‐PAGE results, combinations of Alcalase 2.4 L and Papain were most effective in breaking down the large polypeptides into small peptides. However, DH and SDS‐PAGE are used for initial assessment of integrity of allergen protein structures and further research must be performed to obtain detailed knowledge of immunoreactivity of the hydrolysates. Combinations of Papain and Corolase 7089 showed the best results of foam activity and emulsifying capacity and the lowest bitterness compared to all other hydrolysates. The sensory analysis of all hydrolysates showed similar aroma attributes perception to untreated LPI. Although combinations of Alcalase 2.4 L induced primary undesirable bitter taste. Further studies should address treatments to reduce the bitter taste of the hydrolysates and thus their use as food ingredients.

## CONFLICT OF INTEREST

The authors declare that they have no conflict of interest.

## ETHICAL REVIEW

This study does not involve any human or animal testing.

## INFORMED CONSENT

Written informed consent was obtained from all study participants.
